# Structure Characterization of Zinc Finger Motif 1 and 2 of GLI1 DNA Binding Region

**DOI:** 10.3390/ijms252413368

**Published:** 2024-12-13

**Authors:** Mousheng Wu, Nusrat Jahan, Amanda Sharp, Anwar Ullah, Corinne E. Augelli-Szafran, Sixue Zhang, Rebecca J. Boohaker

**Affiliations:** Scientific Platforms, Southern Research, 2000 9th Avenue South, Birmingham, AL 35205, USA; moushengwu@hotmail.com (M.W.); nursatjahanbuet@gmail.com (N.J.); aksharp@vt.edu (A.S.); aullah@southernresearch.org (A.U.); caugelli-szafran@southernresearch.org (C.E.A.-S.)

**Keywords:** oncology, structural biology, DNA binding protein, GLI1, molecular dynamics simulations, large language model

## Abstract

As a transcription factor, GLI1 plays an important role in cell cycle regulation, DNA replication, and DNA damage responses. The aberrant activation of GLI1 has been associated with cancers such as glioma, osteosarcoma, and rhabdomyosarcoma. The binding of GLI1 to a specific DNA sequence was achieved by five tandem zinc finger motifs (Zif motifs) on the N-terminal part of the molecule. Here, we reported a novel homodimeric crystal structure of Zif1-2. These two Zif motifs are linearized. Namely, Zif2 does not bend and interact with Zif1 of the same molecule. Instead, Zif1 from one molecule interacts with Zif2 from another molecule. The dimer interface of Zif1-2 is unique and different from the conformation of Zif1-2 from the GLI1-DNA co-crystal structure. The dimeric conformation of Zif motifs could represent the native conformation of apo form GLI1 Zif motifs in the cell. The molecular dynamics simulation result of the homodimer, the in silico mutagenesis, and the predicted protease stability of these mutants using a large language model are also presented.

## 1. Introduction

GLI1, a transcription effector at the terminal end of Hedgehog signaling pathways, was originally identified as a highly expressed gene in a malignant glioma [1]. The expression of GLI1 is tightly regulated during embryonic development and tissue differentiation. The aberrant activation of GLI1 has been linked to the promotion of oncogenic activities such as metastasis, DNA damage repair, stemness, and chemotherapeutic resistance in various cancers such as glioma, pancreatic cancer, and colorectal cancer. The increased expression of GLI1 in various cancers is an oncogenic biomarker and a significant therapeutic target for the treatment of multiple cancers [2,3].

Human GLI1 (~1106 amino acids) contains a conserved DNA binding region at the N-terminal region, a SUFU-interacting domain, a nuclear export sequence (NES), a nuclear location signal (NLS), and a transactivation domain (TAD) [4,5,6]. The GLI1 DNA binding region consists of five highly conserved tandem C2H2 zinc finger (Zif) motifs which are responsible for binding to promoters of its downstream target genes [7]. The co-crystal structure of GLI1 Zif motifs with DNA (GLI1-DNA) (PDB ID: 2GLI) was determined [8]. The structure shows that Zif1-3 interacts with the phosphate backbone and contributes to the binding stability and recruitment of co-regulators, while Zif4-5 recognizes the consensus sequence 5′-GACCACCCA-3′ in the promoter regions of target genes, thus regulating gene transcription. In the GLI1-DNA structure, Zif1-2 has less contribution to DNA binding than Zif3-5 [8]. Zif1-2 may open new opportunities for discovering selective inhibitors targeting GLI1. Inhibitors targeting the DNA binding groove of GLI1 (mainly consisting of Zif3-5) may have off-target binding to other DNA binding proteins processing similar DNA binding grooves. Therefore, Zif1-2 of GLI may be a more selective binding region than Zif3-5 for designing and developing direct GLI inhibitors.

Although the Hedgehog signaling pathways, including GLI1, are promising targets for cancer treatment [9], drug discovery efforts directly targeting GLI1 are relatively limited. Direct GLI1 inhibitors such as GANT61 [10], SRI-38832 [3], and JC19 [11] have been reported. However, so far, none of them are in the advanced preclinical or clinical development phase. Of note, the putative binding region of GANT61, SRI-38832, and JC19 is Zif4-5 of GLI. To aid in the discovery of a new generation of GLI1 inhibitors targeting Zif1-2, we determined the crystal structure of the apo form of the first two Zif motifs of GLI1. Interestingly, Zif1 and Zif2 are in a linear configuration and form a unique homodimer in which Zif1 from one molecule interacts with Zif2 from another molecule, and vice versa. The loop regions are also involved in dimerization. This configuration is referred to as the linearized configuration to distinguish it from the bent configuration of Zif1-2 in a DNA-bound state. The bent configuration refers to a configuration in which Zif1 interacts with Zif2 of the same molecule. The *apo* form of Zif1-2 motifs offers a more distinctive structural insight than the DNA-bound GLI1 structure reported before.

## 2. Results

### 2.1. Structure Determination

**Figure 1:**
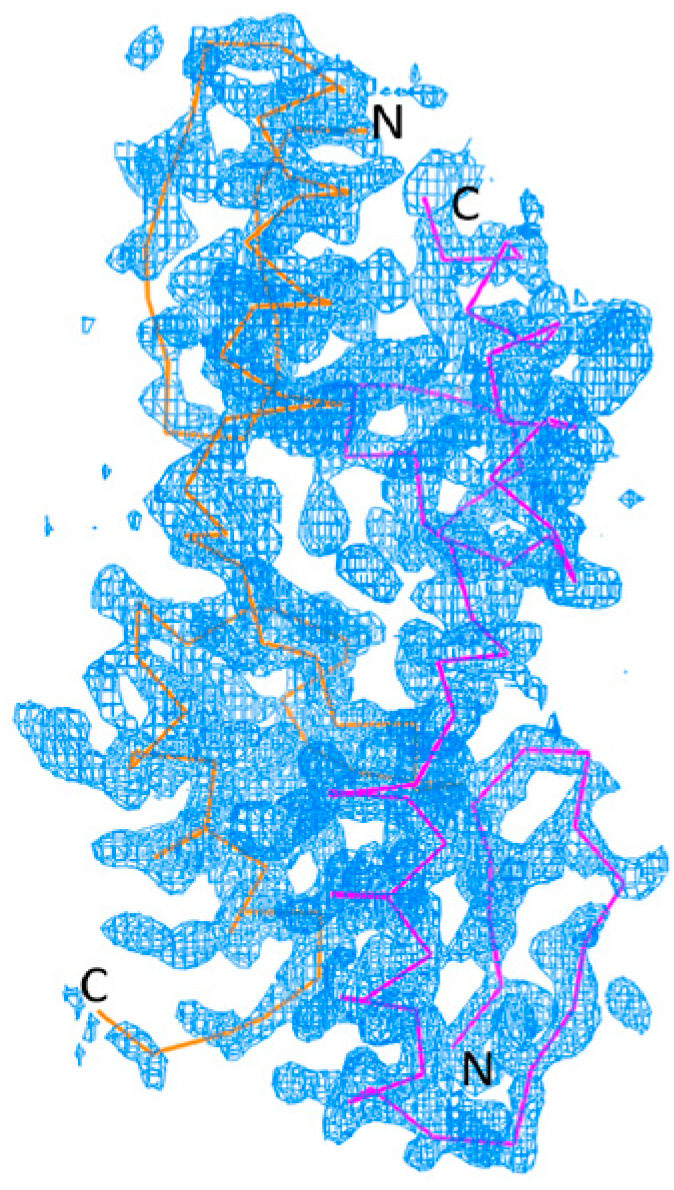
Annealing omit electron density maps with 1.5δ of Zif1-2 models. Pictures were generated using PyMol (The PyMOL Molecular Graphics System, Version 3.0, Schrödinger LLC). Yellow and purple lines indicate the protein backbone of monomer A and B of the dimer crystal structure respectively. Letters N and C mark the beginning of the N-terminus and the end of the C terminus respectively.

The corresponding residues in the structure of GLI1 zinc motifs (PDB ID: 2GLI) were re-numbered to that of the protein sequence from P08151 in the UNIPROT database (www.uniprot.org, accessed on 1 November 1988). For example, E103 in the structure was renumbered to E234. The Zif1-2 (residue 234–302) from 2GLI [8] was used as a model to determine the crystal structure of GLI1 (234–302). The initial model from Phaser [12] was refined by refmac5 [13] and the electron density was checked under COOT [14]. To reduce atom clashes in the initial model, we decided to use ARP/wARP to rebuild the Zif1-2 model automatically using the phase from molecular replacement. The rebuilt model underwent geometry refinement and was then determined to be the final conformation model of the GLI1 Zif1-2 dimer crystal. In the final model, the two Zif1-2 monomers adapted a linear conformation. Namely, the two monomers aligned side-by-side with the N-terminal of one monomer facing the C-terminal of the second monomer (Figure 1).

### 2.2. Overall Structure of Zif1-2

**Figure 2:**
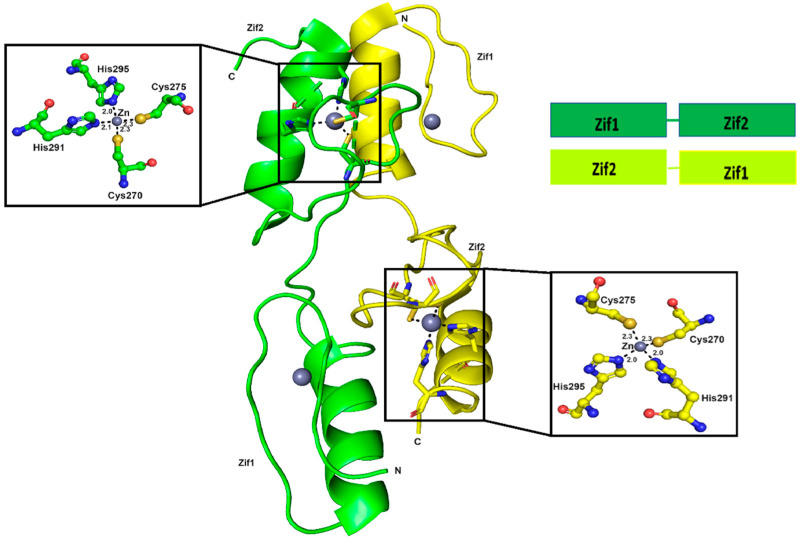
The overall structure of Zif1-2. For simplicity, only two of the four zinc ions are shown in detail. The two monomers are shown in two different colors (green and yellow).

There are two molecules of Zif1-2 in the asymmetric unit in the crystal structure of Zif1-2 (Figure 2). Two zinc ions in each Zif molecule are clearly observed in the crystal structure. Both the zinc ions are tetrahedrally coordinated by two histidines (H291 and H295) and two cysteines (C270 and C275). In the first Zif1-2 molecule, the residues 301–302 are missing in the structure. In the other molecule, the first residue, E234, and residues 297–302 are missing in the structure. Both zinc finger motifs are a typical zinc finger structure which contains two helices. The N-terminal starts with a small loop and the C-terminal ends with a long loop (Figure 2). The structures of both Zif motifs are identical to the structure from 2GLI. Interestingly, Zif1 and Zif2 from both molecules in the crystal structure are linearized, while Zif1 and Zif2 are packed against each other in the GLI1-DNA structure. The Zif1 and Zif2 motifs are opened-up in the structure and form an anti-parallel homodimer with the other linearized Zif1-2 molecule. Zif1 from one molecule is packed against the Zif2 from the other molecule, and vice versa, while in the DNA-bound GLI1 structure, Zif1-2 is a monomer instead of a dimer.

### 2.3. Dimerization of Zif1-2 and Zif1-5

**Figure 3:**
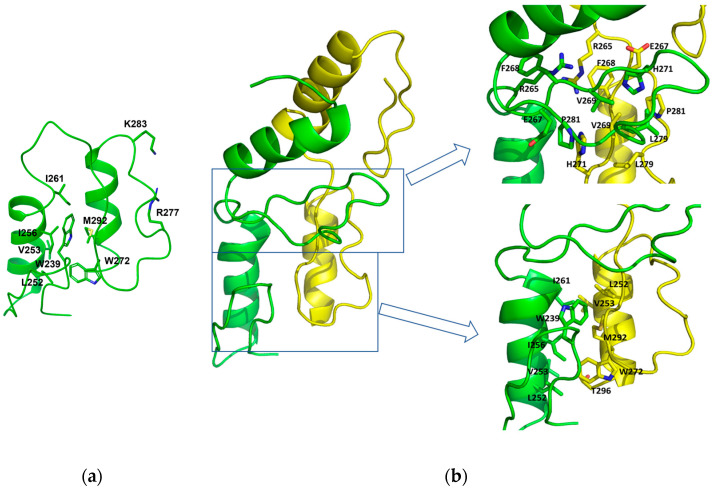
The interface of Zif1 and Zif2: (**a**) Zif1-2 monomer structure from PDB ID 2GLI. (**b**) The new Zif1-2 dimer structure from PDB ID 7T91. The loop between Zif1 and Zif 2 is highlighted in Magenta. Zif1 is shown in green, and Zif2 in yellow. The interface between Zif1 from molecule A (green) and Zif2 from molecule B (yellow) is shown at the bottom on the right panel. The interface contributed to by the loop region and part of β-hairpin from both molecules is shown on the top on the right panel. All residues involved in the interface are represented by sticks.

**Figure 4:**
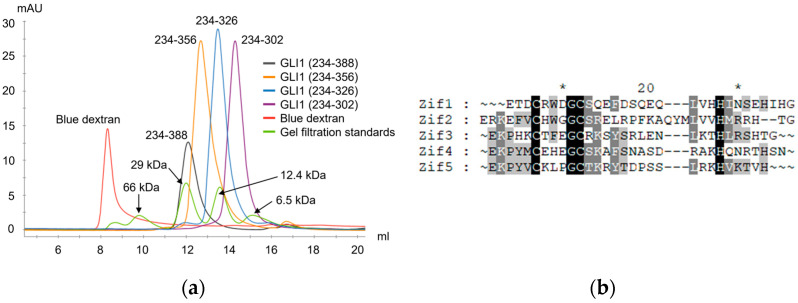
Purification of Zif truncations through size-exclusion chromatography and sequence alignment of all Zif motifs: (**a**) size-exclusion chromatography of all purified Zif truncated proteins; (**b**) sequence alignment of all 5 Zif motifs of GLI1. Sequence alignment was performed using Clustal Omega [15]. Darker color indicates higher sequence consensus with conserved residues among all 5 Zif motifs highlighted in black. Asterisk sign indicates key variant residues.

**Figure 5:**
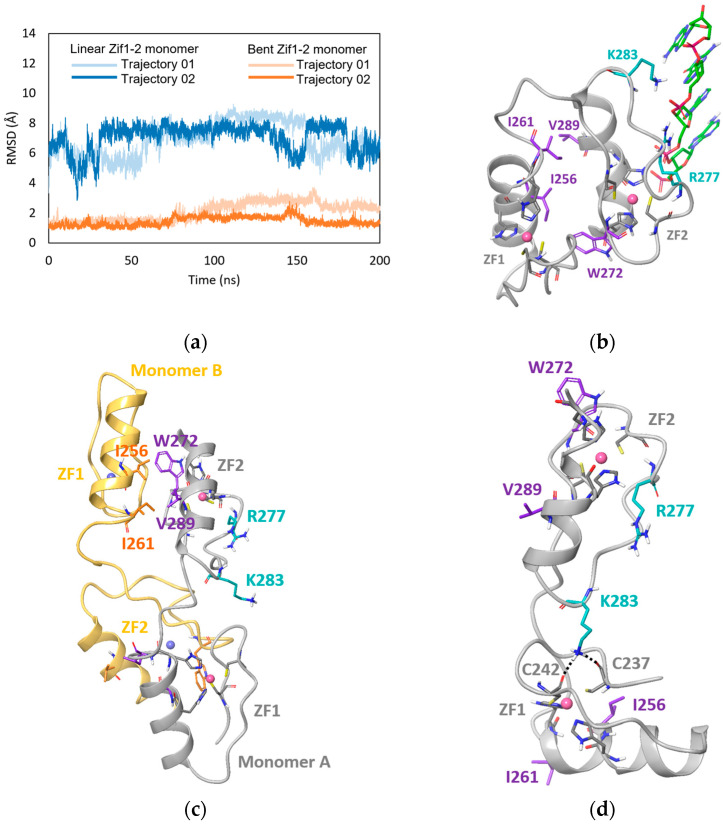
Molecular dynamic simulation studies of Zif1-2: (**a**) RMSD of MD trajectories. (**b**) Bent Zif1-2 from GLI1/DNA crystal complex PDB ID 2GLI. (**c**) Crystal structure of linear Zif1-2 dimer. Monomer A is indicated by gray ribbon, while monomer B is indicated by yellow ribbon. (**d**) Snapshot at 200ns from MD trajectory 1 of linear Zif1-2. Zinc-chelating residues, hydrophobic interface residues, and DNA binding residues are indicated by gray, purple, and turquoise carbons, respectively. Zinc ions are represented by spheres. DNA is indicated by green carbons (not included in MD simulations). Salt-bridge and hydrogen bond interactions are indicated by dashed black lines. Hydrophobic contacts are indicated by dashed magenta lines. Hydrophobic interface residues from monomer B are indicated by orange carbons.

In the monomer GLI1 Zif1-5 structure (PDB ID: 2GLI) [8], Zif1 and Zif2 are connected by a hinge region G(263)ERK(266). Zif1 is in close contact with Zif2, and Zif2 is involved in DNA binding, while Zif1 is not. Hydrophobic residues from Zif1, W239, L252, V253, I256, and I261, form a hydrophobic patch to interact with another hydrophobic patch on Zif2, which is formed by residues W272, L288, V289, and M292 (Figure 3a). On the other side of the Zif2, residues R277, K283, Y286, R293, and R294 interact with the DNA molecule [8] (Figure 3a). In this Zif1-2 dimer crystal structure, the organization of Zif1 and Zif2 is less bent over compared to the 2GLI structure. Although Zif1 and Zif2 are opened-up, the same extensive hydrophobic interactions still contribute to Zif1-2 dimerization. Two major hydrophobic interaction networks, Zif1-Zif2 and the hinge region interaction networks, contribute to the dimerization interface. The same set of residues from the Zif1 and Zif2 interface described in the 2GLI structure are involved in the dimerization, while the hydrophobic patch of Zif1 interacts with that of Zif2 from the other molecule but not from the same molecule (Figure 3b). In the hinge region, residues of the hinge regions (R265) from both the molecules and residues (E267, F268, V269, H271, L279, and P281) of β-hairpins from each Zif2 motif form an extensive hydrophobic interaction, significantly contributing to the dimerization. Each Zif1-2 molecule contributes an approximate 1279 Å^2^ surface area for the dimerization interface. The extensive hydrophobic interaction between the dimers indicates that Zif1-2 can form a stable dimer in the solution. Consistently, the size-exclusion chromatography data (Figure 4a) indicate that the molecular weight of GLI1 Zif1-2 (234–302) was between that of the monomer (~6 kDa) and dimer (~12 kDa). Other different Zif truncations with 3, 4, or 5 Zif motifs, GLI1(234–326), GLI1(234–358), and GLI1(234–388), were also purified for crystallization. Based on size-exclusion chromatography during purification, these Zif truncations also formed a dimer (Figure 4a). GLI1 (234–388, molecular weight ~8 kDa) was eluted in the same elution volume as the standard protein with the molecular weight 12.4 kDa. However, after performing extensive crystallization screenings, we were not able to crystalize other GLI1 Zif truncations besides Zif1-2, implying lower stability of Zif3-5 dimers than Zif1-2 dimers. One possible reason is the lack of hydrophobic patches of Zif3-5 to stabilize their dimer conformation. When we compared the sequences of all five Zif motifs (Figure 4b), the sequence alignment showed that the hydrophobic patches, L(252)VHHI(261) in Zif1 and L(252)VVHM(261), may play a critical role in Zif1-2 dimerization by stabilizing the dimer through intermolecular hydrophobic contact. Such hydrophobic patches are observed in Zif1 and Zif2 but not in the other Zif motifs (Figure 4b). The hydrophobic interface contributing to the dimerization of Zif1-2 mainly consists of I256, I261, W272, and V289 (Figure 5c). More specifically, the Zif1-2 dimer interface involves 7 hydrogen bonds and 117 nonbonded contacts from both monomers (Figure S1). These interactions are contributed by 22 and 20 residues from monomers A and B, respectively. The strength of these dimer interactions was computationally investigated through an in silico mutagenesis study (details in Section 2.5). Of note, there are positive residues (lysine or arginine involved in the recognition of DNA sequences [8]) in other Zif motifs in the location which corresponds to the hydrophobic patches of Zif1 or Zif2.

### 2.4. MD Simulation Study of GLI1 Zif1-2

MD simulations were performed to further study the two different conformational states of GLI1 Zif1-2: the linear monomer conformation from the apo Zif1-2 dimer crystal structure vs. the bent monomer conformation from the 2GLI/DNA co-crystal structure (PDB ID: 2GLI). The root-mean-square deviation (RMSD) calculated from the MD trajectories was used to evaluate the stability of the two conformational states. After a 100 ns simulation, the linear Zif1-2 monomer entered a state with an average RMSD around 7.3 Å whereas the bent Zif1-2 monomer stayed in a state with an average RMSD around 1.7 Å (Figure 5a). This indicated that the linear Zif1-2 monomer was less stable by itself compared to the bent Zif1-2 monomer. Indeed, an intramolecular hydrophobic interface between Zif1 (I256 and I261) and Zif2 (W272 and V289) was presented in the bent monomer in both the crystal structure and MD trajectories, which was supposed to stabilize the conformation (Figure 5b). In contrast, the crystal structure showed that linear Zif1-2 formed an intermolecular hydrophobic interface with I256 and I261 from Zif1 of monomer B and W272 and V289 from Zif2 of monomer A and vice versa (Figure 5c). Such intermolecular hydrophobic contacts were not presented in the MD trajectory of the linear monomer. In addition, during the 100 ns simulation of the linear monomer, the nonpolar residues I256, I261, W272, and V289 faced the solvent phase, not forming intramolecular hydrophobic contacts with other nonpolar residues either. Thus, the monomer in the linearized conformation did not have such a hydrophobic interface for stabilization. Of note, in the *apo* form, the DNA-binding residues R277 and K283 of the linear monomer were able to form random hydrogen bonds with other residues. One example was the hydrogen bonds of K283 with backbones of C237 and C242 (Figure 5d). Overall, the MD simulations indicates that the linear conformation monomer is not stable by itself, and thus, has the need to form dimer for stabilization.

### 2.5. In Silico Mutagenesis

Following the MD simulations, the importance of the hydrophobic interface (I256:I261 and W272:V289) was further examined through in silico mutagenesis. The calculated dimer interaction free energy was used as an implication of the interaction strength between the two monomers (Table 1). The more negative value of the interaction free energy implied a stronger interaction between the two monomers. Taking mutation W272G as an example, it had a loss of 35.66 kcal/mol free energy compared to the wild type (−114.26 vs. −149.92 kcal/mol). This implied that, without the nonpolar side chain of W272 to form hydrophobic contact, the dimer interface was less strong. Similarly, W272N, with a polar side chain not able to form hydrophobic contact, also saw a loss of 31.27 kcal/mol free energy; in other words, it was less stable than the wild-type dimer. Likewise, I256G and I261G resulted in a loss of dimer interaction free energy as well. Interestingly, V289G did not result in a loss of free energy. This might imply that the hydrophobic contact centered around W272 contributed more to dimer stability than the hydrophobic contact centered around V289G. Other mutations also resulted in changes in interaction free energy, indicating that the network of hydrophobic interactions collectively drives Zif1-2 dimerization.

**Figure 6:**
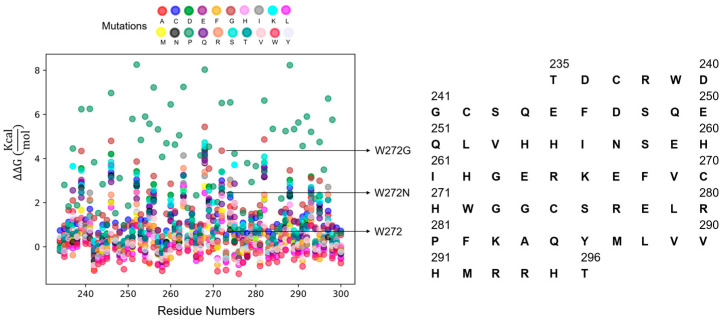
Stability prediction of Zif1-2 mutants using the large language model. The wild-type GLI sequence used for this prediction is listed on the right.

In order to see whether the above mutations of interest would be stable enough to be produced and studied in a wet lab, we leveraged the large language model ProteinBERT [16] to predict the protease susceptibility of all possible mutations (Figure 6). The predictions indicated that the mutations of interest, especially W272G and W272N, were stable mutations. These two mutations were predicted to be even more stable than the wild-type GLI. This may be due to the removal of a protease-recognizable tryptophan residue. It would be warranted in the future to experimentally determine whether W272G and W272N indeed disrupt Zif1-2 dimerization as hypothesized.

## 3. Discussion

**Figure 7:**
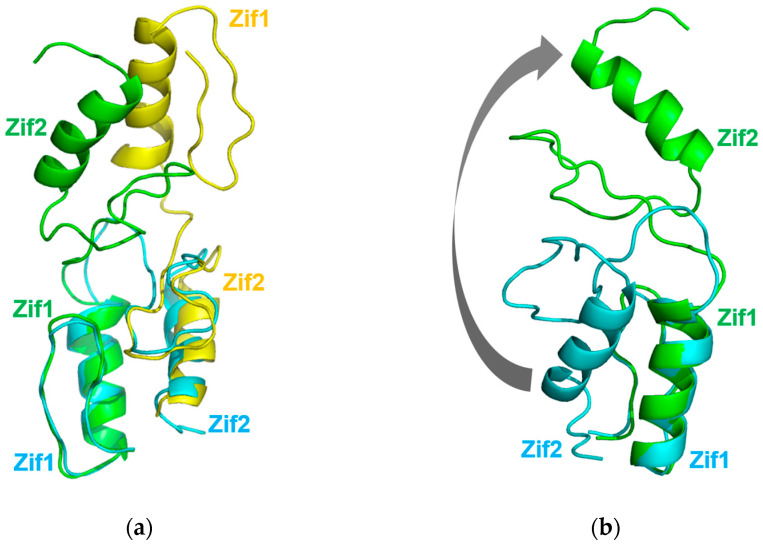
A comparison of the dimeric Zif1-2 structure with the Zif1-2 structure from the 2GLI structure: (**a**) superimposition of Zif1-2 from the 2GLI structure with the dimeric Zif1-2 structure; (**b**) superimposition of Zif1-2 from the 2GLI structure with molecule A of the dimeric Zif1-2 structure. Zif1-2 from the 2GLI structure is shown in cyan. The two molecules of the dimeric Zif1-2 structure are shown in green and yellow, respectively. The arrow shows the moving direction of Zif2 when Zif1-2 forms a linear Zif1-2 conformation.

In the structure of GLI1-DNA in which Zif1-5 is a monomer, Zif1 is in close contact with Zif2 (Figure 3a), adopting a bent conformation. When Zif1-2 from 2GLI is superimposed onto our Zif1-2 dimeric structure, the RMSD of the main chain (residues 234–262) is only 0.855 Å between Zif1 from 2GLI and Zif1 of molecule A in our Zif1-2 structure. However, Zif2 (residues 268–296) from 2GLI is located on Zif2 of molecule B (Figure 7a). From the structure comparison (Figure 7b), the Zif2 from 2GLI needs to rotate about 180 degrees to form a linearized Zif1-2, as seen in the dimeric Zif1-2 structure. The binding of DNA may restrain Zif1-2 from forming a linearized dimer. The Zif1-2 dimer crystal was determined in the absence of DNA. Moreover, the fact that the Zif1-2 apo dimer was observed in size-exclusion chromatography (Figure 4a) indicates that such a dimer is likely to be stable in both the solution and crystal phases.

## 4. Materials and Methods

### 4.1. Molecular Cloning, Protein Expression, and Purification

The yeast SUMO family protein SMT3 gene followed by the TEV cleavage site (SUMO-TEV) was amplified from a pET-His6-SUMO-TEV-LIC plasmid (Addgene #: 29659) via PCR using Phusion high-fidelity DNA polymerase (Thermo Fisher Scientific, Waltham, MA, USA), a forward primer (GGAATTCCATATGTCGGACTCAGAAGTCAATCAAGAAGC), and a reverse primer (CGCGGATCCGGATTGGAAGTACAGGTTTTCCTCGATC). The NdeI restriction site in the forward primer and BamHI site in the reverse primer were underlined. The PCR product was purified using a GeneJET gel extraction kit (Thermo Fisher Scientific) and digested by NdeI and BamHI restriction enzymes. The digested product was purified using a GeneJET PCR purification kit (Thermo Fisher Scientific) and ligated into a pET28a(+) vector, which was digested by NdeI and BamHI enzymes, using T4 DNA ligase (Thermo Fisher Scientific). The ligated product was transformed into NovaBlue *E. coli* competent cells (Novagen), and the plasmids from single colonies were purified by using a GeneJET plasmid miniprep kit (Thermo Fisher Scientific). The insertion of SUMO-TEV was confirmed by DNA sequencing. The plasmid was named the pET28a-SUMO-TEV vector. Human GLI1 truncation containing zinc finger motif 1 and 2 (residue 234–302) were amplified from a longer human GLI1 zinc finger motif construct (residue 232–391) in a pHis-parallel1 vector in our laboratory using the primers in Table 1. The PCR product was digested by BamHI and SalI restriction enzymes and purified as described above. The digested product was ligated to a pET28a-SUMO-TEV vector. The plasmid was prepared from NovaBlue *E. coli* cells (Novagen, Madison, WI, USA) and transformed into *E. coli* Rosetta pLysS competent cells (Novagen) for protein expression. A single colony was inoculated in Luria–Bertani (LB) broth medium and cultured at 37 °C overnight. A 10 mL overnight culture was added to 500 mL of auto-induction medium [9], and the cells were cultured at 37 °C for approximately five hours. The temperature was lowered to 18 °C and the cells were further cultured overnight. The cells were harvested and stocked at −80 °C.

For protein purification, the frozen cell pellet was thawed and resuspended in lysis buffer (20 mM HEPES, pH 7.6, 500 mM NaCl, 0.1 mM TCEP) with 20 mM imidazole, a tablet of EDTA-free protease inhibitors (Thermo Fisher Scientific) and 1 mg/mL lysozyme. The cells were disrupted by sonication, and the cell pellets were spun down at 9500 rpm for 1 h. The supernatant was loaded onto a Ni-NTA column (Cytiva, Marlborough, MA, USA). The Ni-NTA column was then washed stepwise in lysis buffer with 20 mM, 40 mM, and 60 mM imidazole. His-SUMO-GLI (234–302) protein was eluted in lysis buffer with 400 mM imidazole. An amount of 10 mg GST-fused TEV protease solution prepared in the lab was added to the GLI1 elution, and the protein solution was passed through a G-25 desalting column (Cytiva). The protein solution with TEV protease was incubated at 4 °C overnight and then reloaded onto a glutathione column (Cytiva) to remove GST-TEV. The protein solution was diluted in 20 mM Tris pH 8.0 buffer to reach 350 mM NaCl. The GLI1 was further purified by a HiTrap Q HP column (Cytiva) using Buffer A (20 mM Tris, 300 mM NaCl, 0.1 mM TCEP, pH 8.0) and Buffer B (20 mM Tris, 1 M NaCl, 0.1 mM TCEP, pH 8.0). The fractions containing GLI1(234-302) were collected and passed through a Superdex 75 column (Cytiva) in size-exclusion buffer (20 mM HEPES, pH 7.6, 500 mM NaCl, 0.1 mM TCEP). The protein quality from all purification steps was analyzed through SDS-PAGE.

Other Zif truncations, GLI1(234–326), GLI1(234–358), and GLI1(234–388), were cloned using the molecular cloning protocol described above using the forward primer of GLI1(234–302) (Table 2). The reversed primers for GLI1(234–326), GLI1(234–358), and GLI1(234–388) were as follows: GLI1_326SalI (CGCGTCGACTTACGTGTGTGACCGCAGGTGCGTCTTC), GLI1_358SalI (CGCGTCGACTTAATTGGAATGGGTCCGATTCTGGTGC), and GLI1_388SalI (CGCGTCGACTTAACCATGCACTGTCTTGACATGTTTTCG), respectively. All GLI1 Zif truncated proteins were purified using the same protocol as for GLI1(234–302).

### 4.2. Crystallization

Fractions containing the GLI1(234–302) protein from size-exclusion chromatography were collected and concentrated to 5.4 mg/mL using an Amicon 3 kDa molecular-weight cutoff centrifugal concentrator. The concentrated protein was used to set up crystallization screenings using a Phoenix (ArtRobbins Instrument, Sunnyvale, CA, USA) crystallization robot in a 96-well sitting-drop Intelli-plate (ArtRobbins Instrument, Sunnyvale, CA, USA) by mixing 200 nL protein with 200 nL crystallization solutions. The crystallization screen kits in 96-deep wells used for the screening included Crystal Screen, Index, MembFrac, Natrix, PEG/ION, PEGRx, and SaltRx (Hampton Research, Aliso Viejo, CA, USA). The crystallization plates were incubated in a crystallization chamber at 17 °C for at least 3 days. The crystallization plates were examined under a microscope. The initial crystals were grown in Index condition D07 (25% PEG3350, 0.1M Bis-Tris pH 6.5). The crystallization condition was optimized to 29–32% PEG3350, 0.1M Bis-Tris pH 6.5 in a 24-well sitting-drop plate (Hampton Research) under 1 µL protein with 1 µL reservoir solution drop. The crystallization information is summarized in Table 3.

### 4.3. Data Collection and Processing

GLI1(234–302) crystals were cryo-protected in 30% PEG3350, 0.1 M Bis-Tris pH 6.5, and 20% glycerol and frozen in liquid nitrogen. X-ray diffraction data were collected at SER-CAT (Southeast Regional Collaborative Access Team) beamline 22-ID at Advanced Proton Sources (Argonne National Laboratory, Lemont, IL, USA). The data were processed using XDS [17] and scaled using Scala [18]. The data were processed to 2.05 Å and the crystal belonged to space group P65 with the following unit-cell parameters: a = b = 66.72 Å, c = 65.90 Å. The statistics of the data collection and processing are summarized in Table 4.

### 4.4. Structure Solution and Refinement

Initially, the structure of zinc finger motifs 1 and 2 (Zif1-2) without zinc ions (residue 234–302) from the structure of the GLI1 zinc finger motifs (PDB ID: 2GLI) was used as a search model to determine the crystal structure of GLI1(234–302) using Phaser-MR [12]. Two molecules were found during the model search with LLG = 57 and LLG = 146. The initial model then was refined by Refmac5 [13]. The refinement was stuck with a very high Rfree and R factor, 44.78 and 46.92, respectively. Then, the phase from molecular replacement was used to rebuild the model of Zif1-2 using ARP/wARP [19] with the function of automated model building starting from the experimental phases. The R factor of the final ARP/wARP model was 27.79. The model was then refined using Refmac5 to an R factor of 26.38, and an Rfree factor of 28.96. The model was checked and modified in COOT [14]. After several rounds of refinement by Refmac5 and model building in COOT, zinc ions and water molecules were added into the model. The final model was refined to an R factor of 19.8 and an Rfree factor of 22.5 with 99.2% most favored residues and 0.8% allowed residues in the final model checked by Procheck [20]. The statistics of the model refinement are summarized in Table 4. The solved structure was deposited in the PDB Data Bank (www.rcsb.org) with PDB ID: 7T91.

### 4.5. MD Simulation

The bent Zif1-2 monomer used for MD simulations was extracted from the GLI1/DNA co-crystal (PDB ID: 2GLI) and was prepared using the Protein Preparation Wizard [21] in Schrödinger Life Science Suite (Release 2020-1, Schrödinger, LLC: New York, NY, USA). Hydrogen atoms were added to the structure. The PROPKA module [22] in Schrödinger was used to assign the protonation states of the protein residues at pH = 7.4. The linear Zif1-2 monomer was extracted from the *apo* Zif1-2 dimer crystal structure (PDB ID: 7T91) and was prepared for MD simulations using the same procedure as above. MD simulations were performed using the GPU-accelerated AMBER Molecular Dynamics Package [23,24] following the protocol described in the supplementary information. Briefly, an orthogonal solvent box of a TIP3P water model was added to the protein system with a 15 Å thickness on each side. One sodium ion was added to the solvent to neutralize the charge. The system was treated with AMBER force field 12 and a periodic boundary condition. The zinc-chelating motifs were treated with the ZAFF parameters [25,26]. The system underwent seven-step minimization first and then was gradually heated up to a temperature of 300 K under restraints. Subsequently, 10 ns restraint-free equilibration was performed using the NVT ensemble. Once equilibration was reached, a 200 ns MD simulation was carried out using the NVT ensemble. For each system (bent and linear Zif1-Zif2), two independent 200 ns MD trajectories were produced. The temperature and energy of the systems remained stable during the 200 ns simulations (Figure S2). After the MD simulations, the CPPTRJ tool [27] in the AMBER package was used to perform the root-mean-square fluctuation analysis. The crystal structures of bent and linear Zif1-2 were used as the reference frame when calculating the RMSD. The Cα carbon atoms of the protein backbone were used to calculate the RMSD of each frame to the reference frame.

### 4.6. In Silico Mutagenesis and Free Energy Calculation

The Schrödinger Small Molecule Drug Discovery Suite was used to perform the in silico mutagenesis and free energy calculations. After each residue was mutated, molecular mechanics energy minimization was performed on the mutated residue via the Prime module [28] of Schrödinger to optimize its new conformation. Subsequently, the free-energy calculations were performed using the MM-GBSA method implemented in the Prime module.

### 4.7. Large Language AI Model to Predict Protein Stability

To predict the stability upon a single mutation in the Zif1-2 sequence, we used ProteinBERT [16], an open-source LLM that has been pretrained (~106 M known protein sequences to learn protein representation) and fine-tuned (~15 K protein sequences with protease susceptibility assay data) for stability predictions. ProteinBERT exhaustively assessed the impact of every type of amino acid mutation at each position of the given Zif1-2 sequence. There were 1341 single-site mutants for Zif1-2 with a length of 66 AAs (residue numbers 234 to 300 from PDB ID 7T91). The open-source code to run ProteinBERT was obtained from the code repository Github: https://github.com/nadavbra/protein_bert (accessed on 17 December 2023).

## 5. Conclusions

A dimeric Zif1-2 crystal structure is reported here. Zif1-2 forms a unique dimer interface which is totally different from that of the 2GLI structure, though they both use a similar hydrophobic interaction network. The dimer conformation may also be presented in the whole Zif motif (Zif1-5) purified without denaturation and a refolding process, which uses the same Zif1-2 dimer interface. Our MD simulation study showed that the binding of DNA may prevent Zif1-2 dimer formation. Whether the full-length GLI1 molecule in the cell is dimeric warrants further investigation in the future.

## Figures and Tables

**Table 1:** Impact of mutations on Zif1-2 dimerization interaction free energy.

Mutation	Predicted Interaction Free Energy (kcal/mol)
Wild Type	−149.92
W239G	−101.03
W239N	−123.23
L252G	−140.63
L252S	−142.30
V253G	−143.68
V253S	−137.70
I256G	−145.48
I256S	−142.37
I261G	−140.80
I261N	−146.78
I261S	−142.44
W272G	−114.26
W272N	−118.65
W272Y	−142.84
V289G	−151.93
V289T	−160.63
M292G	−133.47
M292N	−135.87
T296S	−147.12

**Table 2:** Macromolecule production information of GLI1(234–302).

Parameter	Value
Source organism	*Homo Sapiens*
DNA source	Laboratory plasmid
Forward primer	CGCGGATCCGAAACTGACTGCCGTTGGGATGGC
Reverse primer	CGCGTCGACTTACTTGTGTGGCTTCTCGCCAGTGTG
Cloning vector	pET28a (+)-SUMO-TEV
Expression vector	pET28a (+)-SUMO-TEV
Expression host	*E. coli* Rosetta pLysS
Sequence of full construct	MGSSHHHHHHSSGLVPRGSHMSDSEVNQEAKPEVKPEVKPETHINLKVSDGSSEIFFKIKKTTPLRRLMEAFAKRQGKEMDSLRFLYDGIRIQADQTPEDLDMEDNDIIEAHREQIGGIEENLYFQSGSETDCRWDGCSQEFDSQEQLVHHINSEHIHGERKEFVCHWGGCSRELRPFKAQYMLVVHMRRHTGEKPHK

**Table 3:** Crystallization of GLI1(234–302).

Parameter	Value
Method	Sitting-drop
Plate type	24-well plate
Temperature (K)	291
Protein concentration (mg/mL)	5.4
Buffer for protein solution	20 mM HEPES, pH = 7.6, 500 mM NaCl, 0.1 mM TCEP
Reservoir solution	29–32% PEG3350, 0.1 M Bis-Tris pH = 6.5
Volume (µL) and ratio of drop	1:1
Volume of reservoir (µL)	500

**Table 4:** Data processing and refinement statistics.

Section	Parameter	Value *
Data collection	Beamline	22-ID, APS
	Processing software	XDS version 2019
	Wavelength (Å)	1.0
	Resolution range (Å)	33.36–2.05 (2.123–2.05)
	Space group	P65
	Unit cell a, b, c (Å)	66.728, 66.728, 65.909
	Unit cell α, β, γ (°)	90, 90, 120
	Total reflections	21,071 (2088)
	Unique reflections	10,536 (1042)
	Multiplicity	2.0 (2.0)
	Completeness (%)	99.88 (100.00)
	Mean *I*/*sigma* (*I*)	17.64 (4.41)
	Wilson B factor (Å^2^)	40.82
	R-merge	0.03403 (0.1799)
	R-means	0.04813 (0.2544)
	R-pim	0.03403 (0.1799)
	CC_1/2_	0.998 (0.899)
Refinement	Reflections used in refinement	10,527 (1044)
	Reflections used for R_free_	509 (56)
	R_work_	0.1985 (0.2403)
	R_free_	0.2258 (0.2982)
	CC_work_	0.948 (0.785)
	CC_free_	0.934 (0.700)
	Number of nonhydrogen atoms	1160
	Macromolecules	1084
	Ligands	4
	Solvent	72
	Protein residues	129
	Bond length (Å)	0.013
	Bond angle (°)	1.60
Ramachandran plot	Ramachandran favored (%)	99.20
	Ramachandran allowed (%)	0.80
	Ramachandran outliers (%)	0.00
	Rotamer outliers (%)	1.69
	Clash score	3.85
	Average B factor (Å^2^)	48.57
	Macromolecules	48.50
	Ligands	39.66
	Solvent	50.03
	Number of TLS groups	2
	PDB ID	7T91

* Values in parentheses are for the highest-resolution shell.

## Data Availability

The data on the crystal structure are openly accessible at the Protein Data Bank with the entry ID 7T91. The link to the data entry is https://www.rcsb.org/structure/7T91 (accessed on 21 December 2022).

## Supplementary Materials

The following supporting information can be downloaded at: https://www.mdpi.com/article/10.3390/ijms252413368/s1.
