# Supplementary material for: Structure Characterization of Zinc Finger Motif 1 and 2 of GLI1 DNA Binding Region
Source: Int J Mol Sci. 2024 Dec 13;25(24):13368. doi: 10.3390/ijms252413368 (PMC11677393; doi:10.3390/ijms252413368)
Supplement: Supplementary file 1 [file ijms-25-13368-s001.zip › Figure S1 and S2.pdf]

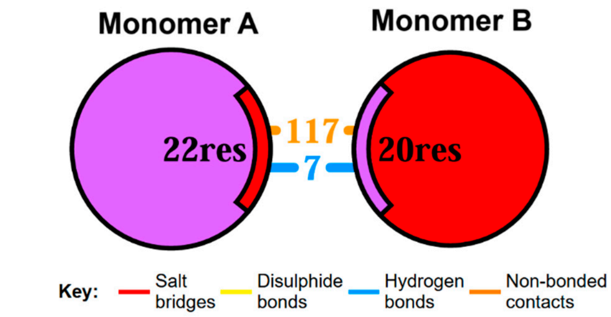

Interface statistics

<table>
<tr><th></th><th>No. of interface residues</th><th>Interface area (Å^2)</th><th>No. of salt bridges</th><th>No. of disulphide bonds</th><th>No. of hydrogen bonds</th><th>No. of non-bonded contacts</th></tr>
<tr><td>Monomer A</td><td>22</td><td>1251</td><td rowspan="2">-</td><td rowspan="2">-</td><td rowspan="2">7</td><td rowspan="2">117</td></tr>
<tr><td>Monomer B</td><td>20</td><td>1311</td></tr>
</table>

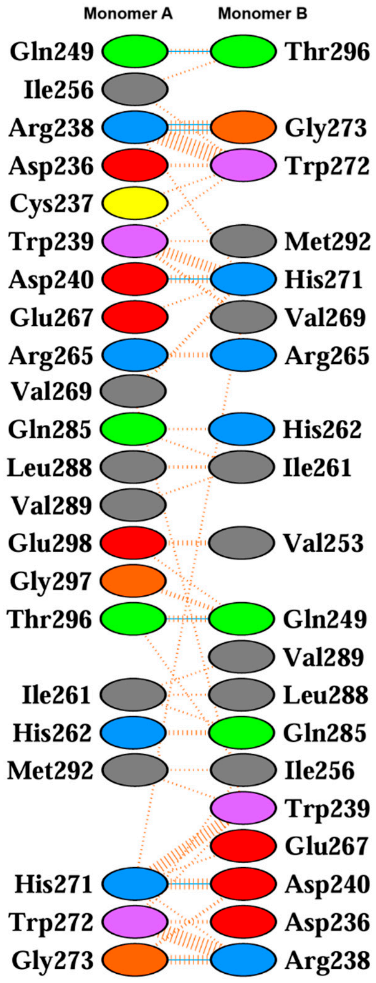

Figure S1. Detailed interactions at the dimer interface of GLI1 Zif1-2 crystal structure depicted using online tool PDBsum (https://www.ebi.ac.uk/thornton-srv/databases/pdbsum).

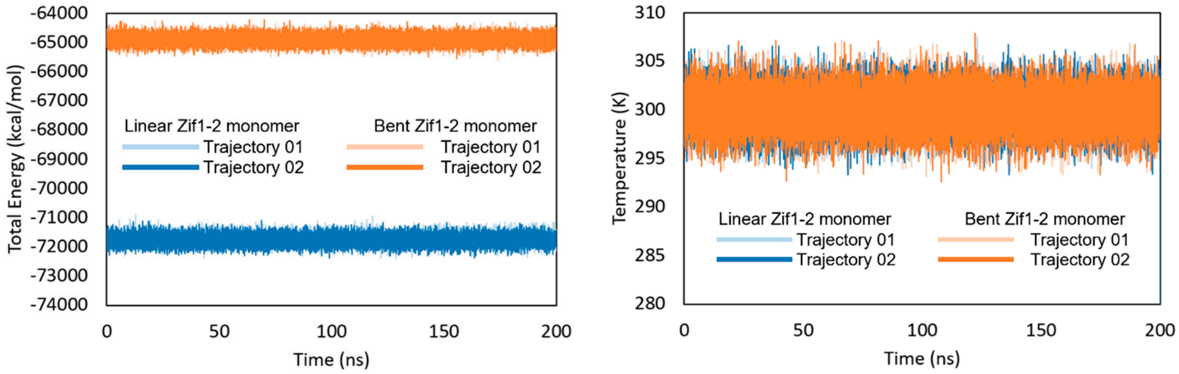

(**a**) (**b**)

Figure S2. Energy and temperature change during molecular dynamics simulations (**a**) Change of total energy; (**b**) Change of temperature of the system. Of note, the energy plots of trajectory 1 and 2 of each system are almost identical. The temperature plots of all 4 trajectories are almost identical.
