# Supplementary material for: Structure Characterization of Zinc Finger Motif 1 and 2 of GLI1 DNA Binding Region
Source: Int J Mol Sci. 2024 Dec 13;25(24):13368. doi: 10.3390/ijms252413368 (PMC11677393; doi:10.3390/ijms252413368)
Supplement: Supplementary file 1 [file ijms-25-13368-s001.zip › Molecular Dynamics Simulation Protocol.pdf]

Molecular Dynamics Simulation Protocol

Below are the 12 input files for the 12 steps performed sequentially using AMBER molecular dynamics simulation software. The exclamation signs in the input files mark the comments.

Step01: *step1 of the 7-step minimization*

```
imin=1,              ! Minimize the initial structure
maxcyc=10000,        ! Maximum number of cycles for minimization
ncyc=5000,           ! Switch from steepest descent to conjugate gradient minimization after ncyc cycles
ntb=1,               ! Constant volume
ntp=0,               ! No pressure scaling
ntf=1,               ! Complete force evaluation
ntc=1,               ! No SHAKE
ntpr=50,             ! Print to mdout every ntpr steps
ntwr=2000,           ! Write a restart file every ntwr steps
cut=10.0,            ! Nonbonded cutoff in Angstroms
ntr=1,
/
Hold the solute fixed
100
RES 1 64
END
END
```

Step02: *step2 of the 7-step minimization*

```
imin=1,             ! Minimize the initial structure
maxcyc=2000,        ! Maximum number of cycles for minimization
ncyc=800,           ! Switch from steepest descent to conjugate gradient minimization after ncyc cycles
ntb=1,              ! Constant volume
ntp=0,              ! No pressure scaling
ntf=1,              ! Complete force evaluation
ntc=1,              ! No SHAKE
ntpr=50,            ! Print to mdout every ntpr steps
ntwr=2000,          ! Write a restart file every ntwr steps
cut=10.0,           ! Nonbonded cutoff in Angstroms
ntr=1,
 /
Fix the heavy atoms
100
FIND
* C * *
* CA * *
* CB * *
* CC * *
* CD * *
* CI * *
* CK * *
* CP * *
SEARCH
RES 1 64
END
Fix the heavy atoms
100
FIND
* CM * *
* CS * *
* CN * *
* CQ * *
* CR * *
* CT * *
* CV * *
* CW * *
SEARCH
RES 1 64
END
Fix the heavy atoms
100
FIND
* C* * *
* CX * *
* CY * *
* CZ * *
* C5 * *
* C4 * *
* C0 * *
* N * *
SEARCH
RES 1 64
END
Fix the heavy atoms
100
FIND
* NA * *
* NB * *
* NC * *
* N2 * *
* N3 * *
* NT * *
```

* N* * *
* NY * *
* O * *
SEARCH
RES 1 64
END
Fix the heavy atoms
100
FIND
* O2 * *
* OW * *
* OH * *
* OS * *
* OP * *
* P * *
* S * *
* SH * *
SEARCH
RES 1 64
END
Fix the heavy atoms
100
FIND
* ZN * *
* S4 * *
SEARCH
RES 1 64
END
END

Step03: *step3 of the 7-step minimization*

```
imin=1,             ! Minimize the initial structure
maxcyc=5000,        ! Maximum number of cycles for minimization
ncyc=2000,          ! Switch from steepest descent to conjugate gradient minimization after ncyc cycles
ntb=1,              ! Constant volume
ntp=0,              ! No pressure scaling
ntf=1,              ! Complete force evaluation
ntc=1,              ! No SHAKE
ntpr=50,            ! Print to mdout every ntpr steps
ntwr=2000,          ! Write a restart file every ntwr steps
cut=10.0,           ! Nonbonded cutoff in Angstroms
ntr=1,
 /
Fix the backbone
100
FIND
CA * * *
C * * *
O * * *
N * * *
SEARCH
RES 1 64
END
END
```

Step04: *step4 of the 7-step minimization*

```
imin=1,             ! Minimize the initial structure
maxcyc=5000,        ! Maximum number of cycles for minimization
ncyc=2000,          ! Switch from steepest descent to conjugate gradient minimization after ncyc cycles
ntb=1,              ! Constant volume
ntp=0,              ! No pressure scaling
ntf=1,              ! Complete force evaluation
ntc=1,              ! No SHAKE
ntpr=50,            ! Print to mdout every ntpr steps
ntwr=2000,          ! Write a restart file every ntwr steps
cut=10.0,           ! Nonbonded cutoff in Angstroms
ntr=1,
 /
Fix the backbone
50
FIND
CA * * *
C * * *
O * * *
N * * *
SEARCH
RES 1 64
END
END
```

Step05: *step5 of the 7-step minimization*

```
imin=1,           ! Minimize the initial structure
maxcyc=5000,      ! Maximum number of cycles for minimization
ncyc=2000,        ! Switch from steepest descent to conjugate gradient minimization after ncyc cycles
ntb=1,            ! Constant volume
ntp=0,            ! No pressure scaling
ntf=1,            ! Complete force evaluation
ntc=1,            ! No SHAKE
ntpr=50,          ! Print to mdout every ntpr steps
ntwr=2000,        ! Write a restart file every ntwr steps
cut=10.0,         ! Nonbonded cutoff in Angstroms
ntr=1,
 /
Fix the backbone
10
FIND
CA * * *
C * * *
O * * *
N * * *
SEARCH
RES 1 64
END
END
```

Step06: *step6 of the 7-step minimization*

```
imin=1,             ! Minimize the initial structure
maxcyc=5000,        ! Maximum number of cycles for minimization
ncyc=2000,          ! Switch from steepest descent to conjugate gradient minimization after ncyc cycles
ntb=1,              ! Constant volume
ntp=0,              ! No pressure scaling
ntf=1,              ! Complete force evaluation
ntc=1,              ! No SHAKE
ntpr=50,            ! Print to mdout every ntpr steps
ntwr=2000,          ! Write a restart file every ntwr steps
cut=10.0,           ! Nonbonded cutoff in Angstroms
ntr=1,
 /
Fix the backbone
1
FIND
CA * * *
C * * *
O * * *
N * * *
SEARCH
RES 1 64
END
END
```

Step07: *step7 of the 7-step minimization*

```
imin=1,            ! Minimize the initial structure
maxcyc=5000,       ! Maximum number of cycles for minimization
ncyc=2000,         ! Switch from steepest descent to conjugate gradient minimization after ncyc cycles
ntb=1,             ! Constant volume
ntp=0,             ! No pressure scaling
ntf=1,             ! Complete force evaluation
ntc=1,             ! No SHAKE
ntpr=50,           ! Print to mdout every ntpr steps
ntwr=2000,         ! Write a restart file every ntwr steps
cut=10.0,          ! Nonbonded cutoff in Angstroms
 /
END
```

Step08: *step1 of the 2-step system heating*

```
imin=0,            ! Molecular dynamics
ntx=1,             ! Positions read formatted with no initial velocities
irest=0,           ! No restart
ntc=2,             ! SHAKE on for bonds with hydrogen
ntf=2,             ! No force evaluation for bonds with hydrogen
tol=0.0000001,     ! SHAKE tolerance
nstlim=2500,       ! Number of MD steps
ntt=3,             ! Langevin thermostat
gamma_ln=1.0,      ! Collision frequency for Langevin thermostat
ig=-1,             ! Random seed for Langevin thermostat
ntpr=100,
ntwr=10000,
ntwx=100,          ! Write to trajectory file every ntwx steps
dt=0.002,          ! Timestep (ps)
nmropt=1,          ! NMR restraints will be read (See TEMP0 control below)
ntb=1,
ntp=0,
cut=10.0,
ioutfm=1,          ! Write a binary (netcdf) trajectory
iwrap=1,           ! Wrap the coordinates
ntwv=-1, ntr=1, restraintmask=':1-64', restraint_wt=10.0,
 /
 &wt
  type='TEMP0',    ! Varies the target temperature TEMP0
  istep1=0,        ! Initial step
  istep2=2500,     ! Final step
  value1=0.0,      ! Initial temp0 (K)
  value2=100.0 /   ! final temp0 (K)
 &wt type='END' /  ! End of varying conditions
```

Step09: *step2 of the 2-step system heating*

```
imin=0,
ntx=5,                 ! Positions and velocities read formatted
irest=1,               ! Restart calculation
ntc=2,
ntf=2,
tol=0.0000001,
nstlim=50000,          ! Number of MD steps
ntt=3,
gamma_ln=1.0,
ntr=1,
ig=-1,
ntpr=100,
ntwr=10000,
ntwx=100,
dt=0.002,
nmropt=1,
ntb=2,                 ! Constant pressure periodic boundary conditions
ntp=2,                 ! Anisotropic pressure coupling
taup=2.0,              ! Pressure relaxation time (ps)
cut=10.0,
ioutfm=1,
iwrap=1,
nmropt=1,
ntwv=-1, ntr=1, restraintmask=':1-64', restraint_wt=10.0,
 /
 &wt
  type='TEMP0',
  istep1=0,
  istep2=50000,
  value1=100.0,
  value2=300.0 /
 &wt type='END' /
```

Step10: *NPT equilibration at 300K for 500ps after system heating*

```
imin=0,
ntx=5,
irest=1,
ntc=2,
ntf=2,
tol=0.0000001,
nstlim=250000,
ntt=3,
gamma_ln=1.0,
temp0=300.0,
ntpr=5000,
ntwr=5000,
ntwx=5000,
dt=0.002,
ig=-1,
ntb=2,
ntp=2,
cut=10.0,
ioutfm=1,
iwrap=1,
nmropt=1,
 /
&wt type='DUMPFREQ', istep1=5000 /
&wt type='END' /
```

Step11: *NVT equilibration at 300K for 10ns after system heating*

```
imin=0,            ! Molecular dynamics
ntx=5,             ! Positions and velocities read formatted
irest=1,           ! Restart calculation
ntc=2,             ! SHAKE on for bonds with hydrogen
ntf=2,             ! No force evaluation for bonds with hydrogen
tol=0.0000001,     ! SHAKE tolerance
nstlim=5000000,    ! Number of MD steps
ntt=3,             ! Langevin thermostat
gamma_ln=1.0,      ! Collision frequency for thermostat
tempi=300.0        ! Initial temperature (K)
temp0=300.0,       ! Simulation temperature (K)
ntpr=5000,         ! Print to mdout every ntpr steps
ntwr=500000,       ! Write a restart file every ntwr steps
ntwx=5000,         ! Write to trajectory file every ntwc steps
ntwv=-1,           ! Write velocity to trajectory files
dt=0.002,          ! Timestep (ps)
ig=-1,             ! Random seed for Langevin thermostat
ntb=1,             ! NVT ensembles
cut=10.0,          ! Nonbonded cutoff (Angstroms)
ioutfm=1,          ! Write binary NetCDF trajectory
iwrap=1,           ! Wrap the coordinates
/
```

Step12: *NVT production run at 300K for 200ns*

```
imin=0,              ! Molecular dynamics
ntx=5,               ! Positions and velocities read formatted
irest=1,             ! Restart calculation
ntc=2,               ! SHAKE on for bonds with hydrogen
ntf=2,               ! No force evaluation for bonds with hydrogen
tol=0.0000001,       ! SHAKE tolerance
nstlim=100000000,    ! Number of MD steps
ntt=3,               ! Langevin thermostat
gamma_ln=1.0,        ! Collision frequency for thermostat
tempi=300.0,         ! Initial temperature (K)
temp0=300.0,         ! Simulation temperature (K)
ntpr=5000,           ! Print to mdout every ntpr steps
ntwr=500000,         ! Write a restart file every ntwr steps
ntwx=5000,           ! Write to trajectory file every ntwc steps
ntwv=-1,             ! Write velocity to trajectory files
dt=0.002,            ! Timestep (ps)
ig=-1,               ! Random seed for Langevin thermostat
ntb=1,               ! NVT ensembles
cut=10.0,            ! Nonbonded cutoff (Angstroms)
ioutfm=1,            ! Write binary NetCDF trajectory
iwrap=1,             ! Wrap the coordinates
 /
```
