# Supplementary figures and images for: Structure Characterization of Zinc Finger Motif 1 and 2 of GLI1 DNA Binding Region
Source: Int J Mol Sci. 2024 Dec 13;25(24):13368. doi: 10.3390/ijms252413368 (PMC11677393; doi:10.3390/ijms252413368)

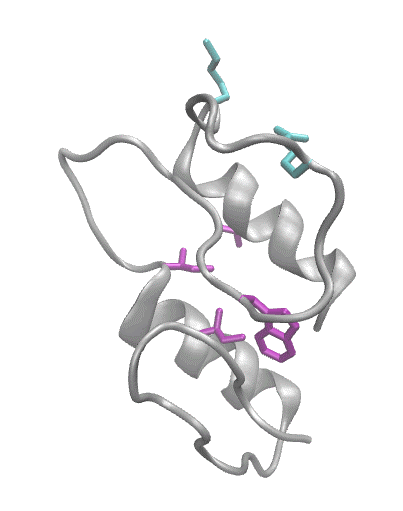

Supplement: Supplementary file 1 [file ijms-25-13368-s001.zip › GLI_Zif1-2_2GLI_trajectory01_movie.gif]

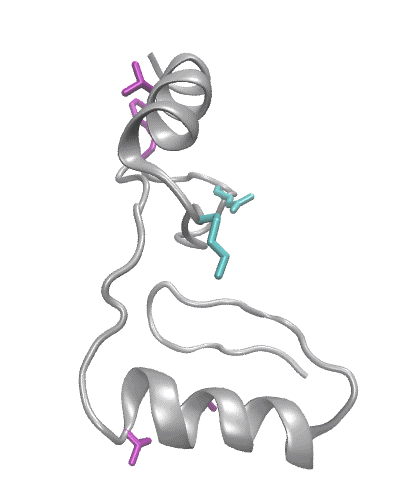

Supplement: Supplementary file 1 [file ijms-25-13368-s001.zip › GLI_Zif1-2_7T91_trajectory01_movie.gif]
